# Supplementary material for: BiSCoT: improving large eukaryotic genome assemblies with optical maps
Source: PeerJ. 2020 Nov 5;8:e10150. doi: 10.7717/peerj.10150 (PMC7649008; doi:10.7717/peerj.10150)
Supplement: Supplemental Information 1 [file peerj-08-10150-s001.pdf]

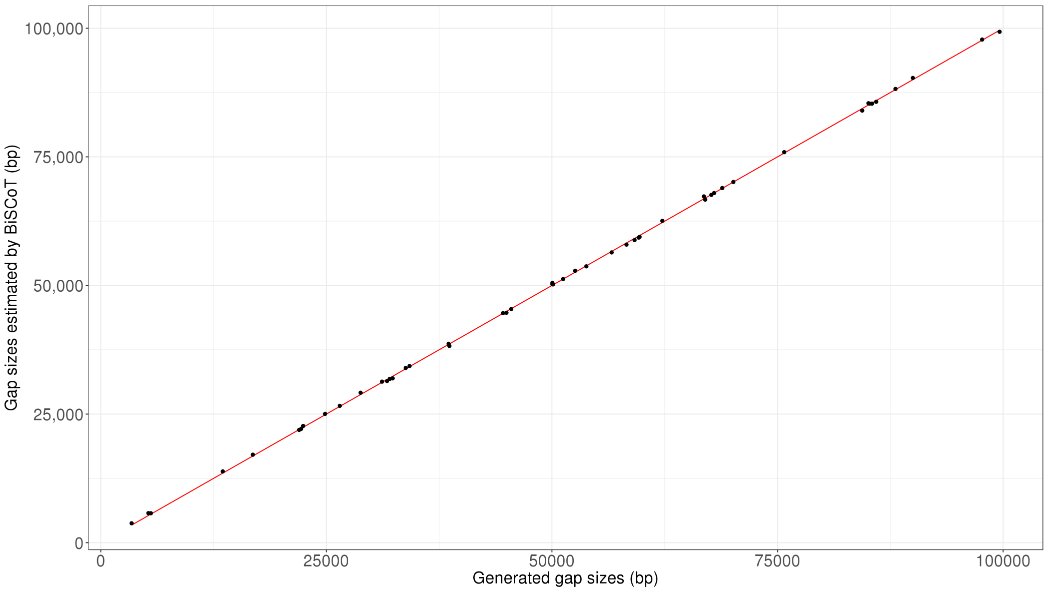

Supplementary Figure 1. Distribution of gap sizes estimated by BiSCoT using the optical maps against the gap sizes introduced in the simulated Human chromosome 1 assembly

	Overlaps	Gaps	Contained contigs
Generated number of events	50	50	5
Cumulative size of events before BiSCoT	2,206,983	2,547,879	1,168,275
Mean size of events before BiSCoT	44,139	50,957	233,655
Min size of events before BiSCoT	278	3,420	215,581
Max size of events before BiSCoT	98,683	99,611	283,879
Remaining number of events after BiSCoT	11	50	0
Cumulative size of events after BiSCoT	30,587	2,549,286	-
Mean size of events after BiSCoT	2680	50,985	-
Min size of events after BiSCoT	278	3,392	-
Max size of events after BiSCoT	5,959	99,792	-

Supplementary Table 1. Metrics of the overlaps, gaps and contained contigs introduced in the simulated Human genome's chromosome 1 assembly before and after applying BiSCoT.

	Simulated contigs	Bionano		BiSCoT	
		Contigs	Scaffolds	Contigs	Scaffolds
Cumulative size	231,215,374	231,215,374	252,023,317	227,870,703	248,278,703
N50	2,429,486	2,363,641	62,178,504	**3,612,465**	61,255,664
L50	46	43	2	**19**	2
N90	**1,677,340**	1,621,176	2,792,747	1,658,986	2,872,288
L90	95	90	10	**55**	9
auN	2,347,866	2,260,168	62,408,635	**4,569,037**	62,312,336
# Ns	0	0	20,789,288	0	20,408,000
NGA50	2,354,935	2,287,164	36,067,187	**2,931,345**	42,227,440
NGA75	1,943,500	1,803,364	2,537,018	**2,034,390**	7,636,847
# misassemblies	**0**	**0**	38	**0**	12

Supplementary Table 2. Metrics of the simulated contigs of the NA12878 chromosome scaffolds and contigs before or after BiSCoT treatment. Bold formatting indicates the best scoring assembly among contigs.

	Nanopore contigs	Bionano		BiSCoT	
		Contigs	Scaffolds	Contigs	Scaffolds
Cumulative size	547,048,580	547,048,579	557,059,506	544,091,840	553,538,342
N50	7,294,619	6,977,344	29,588,784	**12,590,381**	29,458,880
L50	23	23	8	**15**	8
N90	1,304,967	1,183,704	13,953,050	**2,419,034**	13,949,143
L90	85	88	17	**52**	17
auN	9,034,610	8,946,525	29,924,521	**12,646,724**	29,817,651
# Ns	0	0	10,010,927	0	9,446,502
Complete BUSCOs	**1,601 (99.2%)**	**1,601 (99.2%)**	1,598 (99.0%)	**1,601 (99.2%)**	1,600 (99.2%)
Duplicated BUSCOs	235 (14.6%)	235 (14.6%)	232 (14.4%)	**234 (14.5%)**	232 (14.4%)
Missing BUSCOs	10 (0.6%)	**9 (0.6%)**	10 (0.6%)	**9 (0.6%)**	10 (0.6%)

Supplementary Table 3. Metrics of the *Brassica oleracea* HDEM scaffolds and contigs before or after BiSCoT treatment. Bold formatting indicates the best scoring assembly among contigs.

	Nanopore contigs	Bionano		BiSCoT	
		Contigs	Scaffolds	Contigs	Scaffolds
Cumulative size	373,437,357	373,437,357	406,471,180	369,747,840	402,627,824
N50	3,793,063	3,603,274	15,479,745	**5,519,975**	15,275,286
L50	25	26	8	**17**	8
N90	**202,023**	154,330	1,748,645	181,213	1,674,920
L90	264	309	31	**221**	31
auN	5,532,997	5,453,354	18,883,302	**7,237,727**	18,700,050
# Ns	0	0	33,033,823	0	32,879,984
Complete BUSCOs	1,604 (99.4%)	**1,605 (99.5%)**	1,604 (99.4%)	**1,605 (99.5%)**	1,604 (99.4%)
Duplicated BUSCOs	**233 (14.4%)**	235 (14.6%)	234 (14.5%)	**233 (14.4%)**	233 (14.4%)
Missing BUSCOs	**7 (0.5%)**	**7 (0.5%)**	7 (0.5%)	**7 (0.5%)**	7 (0.5%)

Supplementary Table 4. Metrics of the *Brassica rapa* Z1 scaffolds and contigs before or after BiSCoT treatment. Bold formatting indicates the best scoring assembly among contigs.

	Nanopore contigs	Bionano		BiSCoT	
		Contigs	Scaffolds	Contigs	Scaffolds
Cumulative size	518,619,765	518,619,765	526,521,784	517,940,161	525,719,686
N50	4,019,832	2,097,979	36,762,080	**7,987,169**	36,858,856
L50	33	60	6	**24**	6
N90	554,125	292,444	9,697,206	**888,370**	9,721,221
L90	180	310	15	**92**	15
auN	5,390,023	5,285,943	33,951,065	**7,477,787**	33,460,868
# Ns	0	0	7,902,019	0	7,779,525
Complete BUSCOs	1,558 (96.6%)	**1,562 (96.8%)**	1,561 (96.7%)	1,560 (96.6%)	1,559 (96.6%)
Duplicated BUSCOs	69 (4.3%)	**68 (4.2%)**	68 (4.2%)	**68 (4.2%)**	70 (4.3%)
Missing BUSCOs	**34 (2.0%)**	**34 (2.0%)**	34 (2.0%)	**34 (2.0%)**	34 (2.0%)

Supplementary Table 5. Metrics of the *Musa schizocarpa* scaffolds and contigs before or after BiSCoT treatment. Bold formatting indicates the best scoring assembly among contigs.

	Nanopore contigs	Bionano		BiSCoT	
		Contigs	Scaffolds	Contigs	Scaffolds
Cumulative size	652,555,937	652,555,937	665,966,510	649,440,360	662,857,763
N50	2,985,938	2,985,938	31,920,664	**3,969,296**	31,819,818
L50	51	51	10	**42**	10
N90	488,936	485,536	13,186,102	**612,779**	13,076,771
L90	267	261	21	**216**	21
auN	4,975,870	4,969,973	29,298,774	**5,711,235**	29,207,200
# Ns	0	0	13,410,573	0	13,417,403
Complete BUSCOs	1,569 (97.3%)	1,572 (97.4%)	1,576 (97.6%)	**1,576 (97.6%)**	1,573 (97.4%)
Duplicated BUSCOs	**30 (1.9%)**	31 (1.9%)	31 (1.9%)	31 (1.9%)	31 (1.9%)
Missing BUSCOs	26 (1.5%)	24 (1.5%)	23 (1.5%)	**23 (1.5%)**	24 (1.5%)

Supplementary Table 6. Metrics of the *Sorghum bicolor Tx430* scaffolds and contigs before or after BiSCoT treatment. Bold formatting indicates the best scoring assembly among contigs.
