# Supplementary figures and images for: BiSCoT: improving large eukaryotic genome assemblies with optical maps
Source: PeerJ. 2020 Nov 5;8:e10150. doi: 10.7717/peerj.10150 (PMC7649008; doi:10.7717/peerj.10150)

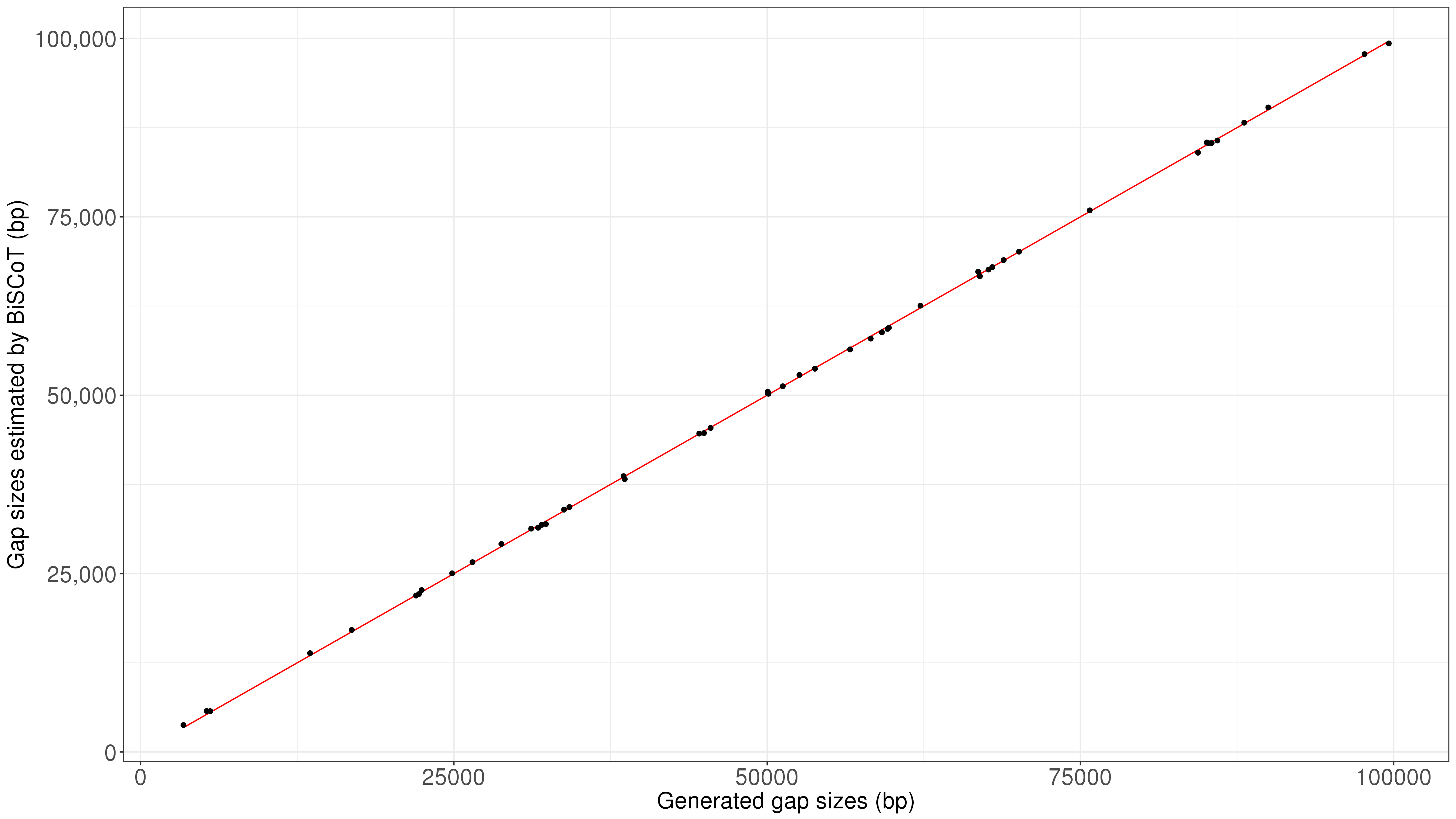

Supplement: Supplemental Information 2 [file peerj-08-10150-s002.png]
